# Effects of benzoic acid in pig diets on nitrogen utilization, urinary pH, slurry pH, and odorous compounds

**DOI:** 10.5713/ab.24.0216

**Published:** 2024-08-23

**Authors:** Seung Bin Yoo, Yoon Soo Song, Siyoung Seo, Sung Woo Kim, Beob Gyun Kim

**Affiliations:** 1 Department of Animal Science, Konkuk University, Seoul 05029, Korea; 2 Animal Environmental Division, National Institute of Animal Science, Wanju 55365, Korea; 3 Department of Animal Science, North Carolina State University, Raleigh, NC 27695, USA

**Keywords:** Benzoic Acid, Odorous Compounds, Pigs, Slurry pH, Urinary pH

## Abstract

**Objective:**

The objective was to investigate the effects of dietary benzoic acid (BA) on nitrogen (N) balance, urinary pH, slurry pH, and odorous compounds in feces and slurry of pigs.

**Methods:**

Twelve barrows with an initial body weight of 56.2±2.6 kg were individually housed in metabolism crates. The animals were allocated to a replicated 6×4 incomplete Latin square design with 12 animals, 6 experimental diets, and 4 periods, resulting in 8 observations per treatment. The basal diet consisted mainly of corn, soybean meal, and rapeseed meal. Benzoic acid was supplemented to the basal diet at 0.5%, 1.0%, 1.5%, 2.0%, or 2.5% at the expense of corn starch. Each period consisted of a 4-day adaptation period and a 24-h collection period for slurry, followed by a 4-day collection period for feces and urine. On day 5, feces and urine were collected for 24 h and mixed to obtain slurry samples.

**Results:**

The daily digested N linearly increased (p<0.05) with increasing BA supplementation. Supplemental BA lowered urinary pH (p<0.001) and slurry pH (p<0.05) in a linear and quadratic manner. In the fecal samples, the concentrations of acetate, propionate, butyrate, and skatole linearly decreased (p<0.05) with supplemental BA. In the slurry samples, the concentrations of butyrate, isovalerate, and skatole linearly decreased (p<0.05) by supplemental BA.

**Conclusion:**

In conclusion, supplemental benzoic acid has the potential to improve nitrogen digestion in a dose-dependent manner for pigs. Additionally, dietary benzoic acid lowers urinary pH, slurry pH, and concentrations of odorous compounds in pig feces and slurry.

## INTRODUCTION

The release of ammonia gas and odorous compounds, such as volatile fatty acids (VFA) and volatile organic compounds, from the pig slurry has been noticed due to environmental problems. The major reason for the volatilization of ammonia gas from urine is the action of urease from bacteria in pig manure [1]. The emitted ammonia gas can lead to the production of fine particulates in the air and eutrophication in ecosystems [2–4]. Odorous compounds, mainly VFA, indole, and phenol compounds, are produced from pig manure through anaerobic fermentation by bacteria that utilize undigested nutrients and endogenous compounds in slurry [5–7]. Eventually, the odorous compounds negatively affect the environment and potentially public health [8].

One of the methods suggested to reduce ammonia emissions from urine is to induce an acidic condition as the activation of urease can be inhibited in acidic urine [9–12]. The supplementation of benzoic acid (BA) in pig diets has been suggested to lower urine pH [13–15]. The ingested BA by pigs is absorbed into the epithelial cell membrane and conjugated with glycine in the liver to be converted into hippuric acid, which is then excreted via urine, resulting in a decrease of urinary pH [13]. In the previous studies, supplemental BA at up to 1.0% [16] or 2.0% [14] resulted in a linear decrease in urinary pH in pigs. However, limited data on the effects of supplemental BA at above 2.0% are available. Moreover, information on the effect of dietary BA on indole and phenol compounds in slurry is not available in spite that supplemental BA potentially reduce the microbes that produce the odorous compounds in pigs [16,17]. Although the excretions of nitrogen (N) from pigs are highly associated with the N balance, the effects of supplemental BA on N digestibility were observed in some studies [17,18] but not in others [16,19]. Therefore, we investigated the effects of supplemental BA at up to 2.5% in pig diets on N balance, urinary pH, slurry pH, and the concentration of VFA, indole compounds, and phenol compounds in pig manure. The supplemental BA was hypothesized to lower urine pH and slurry pH and reduce VFA, indole, and phenol compounds in pig manure potentially by antimicrobial actions.

## MATERIALS AND METHODS

The protocols for the animal experiment were reviewed and approved by the Institutional Animal Care and Use Committee of Konkuk University (KU23055; Seoul, Republic of Korea).

### Animals, experimental design, and experimental diets

Twelve barrows (Landrace×Yorkshire) with an initial body weight of 56.2±2.6 kg were allotted to a replicated 6×4 incomplete Latin square design with 6 diets and 4 periods per square using a spreadsheet method to minimize potential carryover effects [20]. Pigs were individually housed in metabolic crates equipped with a feeder. Six dietary treatments were prepared to contain supplemental BA concentrations at 0%, 0.5%, 1.0%, 1.5%, 2.0%, and 2.5% at the expense of corn starch (Table 1). The experimental diets were formulated to meet or exceed the nutrient requirement estimates suggested by the NRC [21].

### Feeding and sample collection

The experimental diets were provided to pigs at a daily amount of 3.0 times the estimated energy requirement for maintenance (i.e., 197 kcal of metabolizable energy per kg body weight^0.60^; [21]). The daily feed allotment was divided into two equal meals and fed to pigs at 0800 and 1700 h. Water was freely available at all times. An experimental period consisted of a 4-day adaptation period [22], followed by a 24-h collection period for making slurry samples, and a 4-day collection period for total feces and urine collection. On day 5, feces and urine samples were collected during the 24-h period and mixed at a ratio of feces weight to urine weight to make slurry samples. Feces were collected according to the marker-to-marker procedure using chromic oxide as a marker, and urine was collected in a plastic container from 1000 h on day 6 to 1000 h on day 10 [23]. Urine was weighed and collected twice daily at 1000 h and 1900 h. The urine pH was immediately measured using a pH meter (PM-2, CAS Inc., Yangju, Korea) after weighing the urine. All feces and urine samples were stored at –20°C immediately after collection.

### Volatile fatty acid analyses

For the VFA analyses in the feces and the slurry, 1 mL of 25% meta-phosphoric acid solution (Sigma-Aldrich, St. Louis, MO, USA) and 0.05 mL of saturated mercury(II) chloride solution (Sigma-Aldrich, USA) in a 15-mL plastic tube were prepared to mix with 5 g of feces and 5 mL of slurry. The mixed solution was then centrifuged at 3,134×g for 20 min at 20°C. One milliliter of supernatant was subsequently centrifuged at 13,800×g for 10 min and filtered through a 0.2 μm filter (Whatman, Uppsala, Sweden). Filtrates were transferred to 2.0-mL gas chromatography vials (Agilent, Santa Clara, CA, USA). The concentration of VFA was analyzed using a gas chromatography (6890N; Agilent, USA) equipped with an HP-INNOWax column (30 m×0.25 mm×0.25 μm; Agilent, USA) and a flame ionization detector. The sample injection volume was 0.2 μL with a 10:1 split ratio. The oven temperature was initially set at 80°C for 2 min, increased to 120°C at the rate of 20°C/min, then to 205°C at the rate of 10°C/min, and finally held at 205°C for 2 min. The injection and detection ports were maintained at 250°C.

### Phenol and indole analyses

For phenols and indoles, slurry samples were centrifuged at 3,134×g for 20 min at 20°C, and then 4 mL of supernatant was mixed with 4 mL of chloroform (Merck, Darmstadt, Germany) and 60 μL of 4 *M* sodium hydroxide solution (Sigma-Aldrich, USA) in a 20-mL glass vial. The mixture was centrifuged at 3,134×g for 20 min at 20°C, and the chloroform layer was transferred to a 2.0-mL gas chromatography vial (Agilent, USA). Phenols and indoles were analyzed using a gas chromatography (6890N; Agilent, USA) equipped with a DB-1 column (30 m×0.25 mm×0.25 μm; Agilent, USA) and a flame ionization detector. The sample injection volume was 2.0 μL with a 5:1 split ratio. The oven temperature was initially set at 40°C for 5 min, increased to 230°C at the rate of 10°C/min, and then held at 230°C for 2 min. The injection and detection ports were maintained at 250°C.

### Chemical analyses

All chemical components were determined according to the AOAC [24]. The feces samples were dried in a forced-air drying oven at 55°C until a constant weight was achieved and ground before analysis. Dry matter (DM; 135°C for 2 h; method 930.15), ash (method 942.05), and ether extract (method 920.39) in the ingredients and diets were analyzed. Gross energy in the ingredient and experimental diet samples was determined using bomb calorimetry (Parr 6400; Parr Instruments Co., Moline, IL, USA). Samples of ingredient, diet, feces, and urine were analyzed for N (method 990.03). Crude protein was calculated by multiplying N by 6.25. Amylase-treated neutral detergent fiber (method 2002.04) concentration in the ingredient and diet samples was analyzed with heat-stable amylase, and acid detergent fiber (method 973.18) in the ingredients and diets was also determined.

### Calculation

The apparent total tract digestibility (ATTD) of DM and N was calculated with the following equations [25]:


$$\begin{array}{l} \text{ATTD of DM }(\%)=[({\text{DM}}_{\text{intake}}-{\text{DM}}_{\text{feces}})/{\text{DM}}_{\text{intake}}]\times 100\\ \text{ATTD of N }(\%)=[({\text{N}}_{\text{intake}}-{\text{N}}_{\text{feces}})/{\text{N}}_{\text{intake}}]\times 100 \end{array}$$

where the DM_intake_ and DM_feces_ represent the amount of DM intake (g/d) and the amount of fecal DM output (g/d), respectively, and where N_intake_ and N_feces_ represent the amount of N intake (g/d) and the amount of fecal N output (g/d), respectively.


$$\begin{array}{l} \text{N retention rate as }\%\;\text{of ingested }(\%)=({\text{N}}_{\text{intake}}-{\text{N}}_{\text{feces}}-{\text{N}}_{\text{urine}})/{\text{N}}_{\text{intake}}\times 100\\ \text{N retention rate as }\%\;\text{of digested }(\%)=({\text{N}}_{\text{intake}}-{\text{N}}_{\text{feces}}-{\text{N}}_{\text{urine}})/({\text{N}}_{\text{intake}}-{\text{N}}_{\text{feces}})\times 100 \end{array}$$

where the N_urine_ represents the amount of urinary N output (g/d).

### Statistical analyses

Experimental data were analyzed using the MIXED procedures of SAS (SAS Inst. Inc., Cary, NC, USA). The model included diet as the fixed variable and replication, animal within replication, and period within replication as the random variables. Least squares means were calculated for each dietary treatment. Orthogonal polynomial contrasts were conducted to analyze linear and quadratic effects of BA supplementation on N balance in pigs. Additionally, urinary pH data were analyzed using the repeated measure analysis procedure over the collection period [26]. Dietary treatments, days, and the interaction between the main effects were included in the statistical model as fixed effects. Additionally, polynomial contrasts were conducted to determine the linear and quadratic effects of BA and days. For all statistical analyses, each pig was the experimental unit, and statistical significance and tendency were declared at p<0.05 and 0.05≤ p<0.10, respectively.

## RESULTS

All the pigs remained in good health throughout the experiment and readily consumed their daily feed allowance. The ATTD of N tended to linearly increase (p = 0.054) and digested N linearly increased (p<0.001) with increasing dietary BA at up to 2.5% (Table 2). Supplemental BA lowered urinary pH (p<0.001) and slurry pH (p<0.05) in a linear and quadratic manner (Table 3). Additionally, urinary pH increased (p<0.001) as the time passed for 5 days.

In the fecal samples, the concentrations of acetate, propionate, butyrate, and skatole linearly decreased (p<0.05) by supplemental BA, and the isovalerate concentration tended to decrease linearly (p = 0.099) as dietary BA increased (Table 4). In the slurry samples, the concentrations of butyrate, isovalerate, and skatole linearly decreased (p<0.05) as dietary BA increased, and the concentrations of acetate and propionate tended to decrease linearly (p<0.10) as dietary BA increased.

## DISCUSSION

Benzoic acid is an aromatic carboxylic acid with a carboxyl group attached to a benzene ring [27]. The dietary BA is absorbed into the intestinal epithelial cells of pigs, converted into hippuric acid by conjugating with glycine, and consequently excreted via urine as hippuric acid, lowering urine pH [28]. Experiments with the BA dose over 2.0% for pig diets are scarce and the measurements for odorous compounds were mostly limited to lactic acids and VFA in the slurry. The present work aimed to measure the effects of supplemental BA at up to 2.5% in pig diets on urinary pH, slurry pH, and the concentration of VFA, indole compounds, and phenol compounds in pig manure.

The reason for the linear increase in N digestibility with supplemental BA is likely that dietary BA has the potential to enhance digestive enzyme activity by lowering gastrointestinal pH [17,18,29] and to improve morphology of the proximal gastrointestinal tract [18,30], acting as a type of organic acids, consequently increasing nutrient digestibility in nursery pigs [17,18,29,31–33]. It should be notable that previous BA studies used nursery pigs whereas 56-kg pigs were used in the present work, which may be a potential reason for the relatively limited effects of BA on N digestibility in the present experiment. Although anecdotal, Murphy et al [19] failed to find the effects of BA at 3% on N digestibility in 64-kg pigs. The effects of BA on N digestibility are likely to be more apparent in pigs at an early growth stage. On the other hand, Kluge et al [16] suggested that dietary BA would have limited effects on digesta acidification even in nursery pigs as BA is not easily dissociated to release the hydrogen ions in the stomach. The effects of BA on nutrient digestibility of pigs are also likely to be associated with the ingredients used in the diets.

The effects of increasing dietary BA on lowering urinary and slurry pH in pigs observed in the present work are consistent with the observations in previous studies [14,16,34,35]. However, the magnitude of BA for lowering urinary pH in the present work was less than that reported in the literature considering the dose of BA. Sauer et al [14] reported that urinary pH decreased from 7.32 to 5.32 when pigs were fed a diet supplemented with 2% BA. Bühler et al [34] also reported that urinary pH decreased from 7.77 to 6.76 when pigs were fed a diet supplemented with 1% BA. This discrepancy may be attributed to the increase in urinary pH over time during the collection periods in the present study. The reason for this increase in urinary pH over time remains unclear but might be partially explained by the potential toxicity of dietary BA. Shu et al [36] suggested that BA accumulated in the liver and the kidneys when pigs were fed diets containing 0.5%, 2.5%, or 5.0% BA for 56 days compared with those fed the control diet. The accumulated BA can damage the organs, particularly with high doses and long-term intake of BA [36]. Therefore, the damaged liver and kidneys in the present work might not have been able to metabolize BA or excrete BA residues during the 52-day experimental period.

Additionally, the different methods for collecting pig urine among the experiments may also contribute to the magnitude of the decrease in urinary pH. In previous studies, urine samples were collected directly from the bladder after euthanizing pigs [15,16] or through an equipped catheter [19,35], which may prevent microbial fermentation and allow for a lower urinary pH. In contrast, urine samples were collected in the bucket placed under the metabolism crates 2 times per day during the collection periods in the present experiment. This procedure potentially increases the exposure of urine to external factors such as air and microbial action. However, this does not fully explain the increased urinary pH over time during the collection period. Further research is warranted to identify the long-term effects of BA on urinary pH.

The odorous compounds including VFA, indole compounds, and phenolic compounds are end-products resulting from the large-intestinal fermentation of carbohydrates, branched-chain amino acids, and aromatic amino acids [37,38]. The reduction in short-chain fatty acid concentrations in the feces and slurry by dietary BA observed in the present work is consistent with Halas et al [17] who reported that the addition of 0.5% BA to pig diets reduced odorous compound concentrations such as butyrate and isovalerate in the cecal digesta. The effects of BA on the reduction of VFA may be explained by the reduced population of *Escherichia coli* and lactic acid bacteria in the gastrointestinal tract that produce short-chain fatty acids [16,39]. Additionally, the reduction in the abundance of *Megasphaera* and *Streptococcus* strains in the gut microbiota was observed in the mice by supplemental BA at 0.5% [40]. The reduction of skatole in fecal and slurry samples in the present study can be also explained by the effects of dietary BA on lactic acid bacteria. The strain of the *Lactobacillus* genus, which belongs to the group of lactic acid bacteria, can produce skatole using indole compounds converted from unabsorbed tryptophan [41]. Therefore, the production of skatole is likely to be reduced by supplemental BA because dietary BA decreased the number of *Lactobacillus* genus in the intestine of pigs.

## CONCLUSION

In the present study, dietary benzoic acid at up to 2.5% increased nitrogen digestibility and decreased urinary pH, slurry pH, and the concentrations of odorous compounds in feces and slurry of pigs. However, the extent of the urinary pH reduction by dietary benzoic acid decreased over time during the experimental period.

## Figures and Tables

**Table 1:** Ingredient and chemical composition of experimental diets, as-fed basis

Item	Benzoic acid (%)					

	0.0	0.5	1.0	1.5	2.0	2.5
Ingredient (%)						
Ground corn	70.1	70.1	70.1	70.1	70.1	70.1
Soybean meal (45.1% crude protein)	14.0	14.0	14.0	14.0	14.0	14.0
Rapeseed meal	10.0	10.0	10.0	10.0	10.0	10.0
Soybean oil	1.0	1.0	1.0	1.0	1.0	1.0
L-Lys·HCl (78.8%)	0.25	0.25	0.25	0.25	0.25	0.25
L-Thr (99.0%)	0.04	0.04	0.04	0.04	0.04	0.04
Dicalcium phosphate	0.8	0.8	0.8	0.8	0.8	0.8
Ground limestone	0.7	0.7	0.7	0.7	0.7	0.7
Salt	0.3	0.3	0.3	0.3	0.3	0.3
Vitamin-mineral premix^1)^	0.3	0.3	0.3	0.3	0.3	0.3
Corn starch	2.5	2.0	1.5	1.0	0.5	-
Benzoic acid	-	0.5	1.0	1.5	2.0	2.5
Calculated chemical composition						
Metabolizable energy (kcal/kg)	3,339	3,319	3,300	3,280	3,260	3,240
Standardized ileal digestible Lys (%)	0.85	0.85	0.85	0.85	0.85	0.85
Calcium (%)	0.59	0.59	0.59	0.59	0.59	0.59
STTD phosphorus (%)	0.27	0.27	0.27	0.27	0.27	0.27
Analyzed chemical composition						
Dry matter (%)	88.3	88.1	87.7	87.2	87.2	86.3
Gross energy (kcal/kg)	3,838	3,854	3,871	3,873	3,899	3,881
Crude protein (%)	16.8	16.6	16.6	16.6	16.6	16.9
Ether extract (%)	3.9	4.4	4.3	4.4	4.6	4.5
Ash (%)	4.9	4.8	4.9	4.7	4.7	4.6
Neutral detergent fiber (%)	11.2	11.9	11.4	11.3	10.8	11.5
Acid detergent fiber (%)	4.4	4.3	4.3	4.1	3.6	4.4

STTD, standardized total tract digestible.

1) Provided the following quantities per kilogram of complete diet: vitamin A as retinyl acetate, 18,000 IU; vitamin D_3_ as cholecalciferol, 3,600 IU; vitamin E as DL-α-tocopheryl acetate, 60 mg; vitamin K as menadione nicotinamide bisulfite, 4.5 mg; thiamin as thiamine mononitrate, 4.5 mg; riboflavin, 7.5 mg; pyridoxine as pyridoxine hydrochloride, 4.5 mg; vitamin B_12_, 0.06 mg; D-pantothenic acid as D-calcium pantothenate, 30 mg; folic acid, 1.5 mg; niacin as nicotinamide, 45 mg; biotin, 0.3 mg; Co as cobaltous carbonate, 0.75 mg; Cu as copper sulfate, 120 mg; Fe as iron sulfate, 120 mg; I as calcium iodate, 0.75 mg; Mg as magnesium oxide, 60 mg; Mn as manganese sulfate, 60 mg; Se as sodium selenite, 0.3 mg; Zn as zinc sulfate, 90 mg.

**Table 2:** Apparent total tract digestibility (ATTD) of dry matter (DM) and nitrogen (N) balance in pigs fed the experimental diets^1)^

Item	Benzoic acid (%)						SEM	p-value	
	
	0.0	0.5	1.0	1.5	2.0	2.5		Linear	Quadratic
DM intake (kg/d)	2.0	2.0	2.0	2.0	2.0	2.0	0.1	0.117	0.044
N intake (g/d)	61.0	60.8	61.9	61.0	62.4	63.2	2.3	<0.001	0.013
Fecal DM output (kg/d)	0.23	0.24	0.25	0.23	0.23	0.24	0.01	0.635	0.723
ATTD of DM (%)	89.4	89.0	89.0	89.8	89.7	89.4	0.41	0.291	0.985
Fecal N output (g/d)	7.46	7.61	7.89	7.03	7.14	7.34	0.39	0.211	0.852
ATTD of N (%)	87.7	87.5	87.3	88.5	88.6	88.4	0.5	0.054	0.759
Urine output (kg/d)	4.4	4.6	4.6	4.4	4.3	4.8	0.5	0.676	0.804
Urinary N output (g/d)	21.9	20.8	22.8	21.7	23.1	22.7	2.3	0.265	0.904
Digested N (g/d)	53.5	53.2	54.1	53.9	55.2	55.8	2.02	<0.001	0.100
Retained N (g/d)	31.6	32.5	31.3	32.3	32.0	33.1	2.7	0.372	0.585
N retention (% of ingested)	51.5	52.9	50.4	52.7	51.4	52.4	3.2	0.857	0.845
N retention (% of digested)	58.9	60.4	57.9	59.6	58.2	59.3	3.8	0.854	0.888

SEM, standard error of the means.

1) Each least squares mean represents 8 observations.

**Table 3:** Urinary and slurry pH of pigs fed the experimental diets^1)^

Item	Benzoic acid (%)						Mean	SEM	p-value^2)^		
	
	0.0	0.5	1.0	1.5	2.0	2.5			Day	Dose	Day×Dose
Urinary pH											
Day 1	8.61	8.55	8.26	7.44	6.77	6.44	7.68	0.19	<0.001	<0.001	0.195
Day 2	8.83	8.84	8.61	8.11	7.68	7.24	8.22				
Day 3	8.74	8.80	8.68	8.33	7.70	7.24	8.25				
Day 4	8.85	8.87	8.77	8.48	8.16	7.86	8.50				
Day 5	8.78	8.83	8.74	8.34	8.02	7.56	8.38				
Mean	8.76	8.78	8.61	8.14	7.66	7.27					
Slurry pH	8.68	8.67	8.61	8.34	8.38	7.69		0.13		<0.001	

SEM, standard error of the means.

1) Each least squares mean represents 8 observations.

2) Day, collection day; Dose, supplementation of benzoic acid in diet; Day×Dose, interaction between collection day and supplementation of benzoic acid in diet. The day effect on urinary pH was linear (p<0.001) and quadratic (p<0.001). The dose effect on urinary pH was linear (p<0.001) and quadratic (p<0.001). The dose effect on slurry pH was linear (p<0.001) and quadratic (p = 0.016).

**Table 4:** Volatile fatty acids (VFA), phenol compounds, and indole compounds in feces and slurry from pigs fed the experimental diets^1)^

Item (mg/L)	Benzoic acid (%)						SEM	p-value	
	
	0.0	0.5	1.0	1.5	2.0	2.5		Linear	Quadratic
VFA in feces									
Acetate	3,694	4,160	3,801	3,677	3,280	3,415	193	0.011	0.315
Propionate	1,543	1,598	1,566	1,518	1,410	1,351	98	0.016	0.194
Isobutyrate	209	218	202	176	184	191	19	0.126	0.494
Butyrate	855	887	765	849	686	632	80	0.009	0.357
Isovalerate	382	395	378	323	324	348	33	0.099	0.642
Valerate	270	261	264	250	249	247	27	0.362	0.931
Phenol compounds in feces^2)^									
*p*-Cresol	9.59	18.48	10.44	9.55	8.21	8.42	3.44	0.172	0.760
Indole compounds in feces									
Indole	3.04	2.00	3.11	1.72	2.87	2.42	0.79	0.759	0.665
Skatole	5.63	5.01	4.76	3.38	3.36	3.40	0.99	0.038	0.646
VFA in slurry									
Acetate	957	1,033	933	846	824	830	131	0.056	0.960
Propionate	186	215	191	154	152	167	25	0.051	0.825
Isobutyrate	25.2	29.8	23.7	17.4	20.9	22.4	4.0	0.115	0.346
Butyrate	97.5	120.4	99.9	82.9	74.4	81.2	15.1	0.038	0.873
Isovalerate	50.5	57.4	46.5	33.5	39.4	40.6	7.4	0.034	0.369
Valerate	32.5	34.5	32.8	25.4	27.7	30.2	4.8	0.211	0.537
Phenol compound in slurry									
Phenol	2.54	2.94	3.74	2.80	2.85	3.75	0.55	0.254	0.804
*p*-Cresol	75.4	79.8	83.5	67.5	69.6	67.6	10.8	0.252	0.646
Indole compound in slurry									
Indole	0.23	0.54	0.47	0.20	0.40	0.35	0.12	0.922	0.731
Skatole	0.78	0.85	0.77	0.48	0.54	0.58	0.13	0.033	0.624

SEM, standard error of the means.

1) Each least squares mean represents 8 observations.

2) Phenol was not detected in the fecal samples.
